# Integrated Stress Physiology and Mitigation Strategies for Heat Stress in Layer Chickens—Review

**DOI:** 10.3390/ani16071001

**Published:** 2026-03-24

**Authors:** Peter Ayodeji Idowu, Caroline Chauke, Takalani J. Mpofu

**Affiliations:** Department of Animal Sciences, Faculty of Science, Tshwane University of Technology, Pretoria 0001, South Africa

**Keywords:** laying hens, poultry physiology, oxidative stress, immune response, nutritional mitigation strategies, environmental management, egg production

## Abstract

Heat stress is a major constraint to global egg production, as rising temperatures increasingly challenge the physiological limits of commercial layer chickens. This review integrates current advances in stress physiology to demonstrate that heat stress is not merely a thermoregulatory problem but a multi-systemic disruption involving neuroendocrine overload, metabolic imbalance, oxidative damage, immune suppression, and gastrointestinal barrier breakdown. These interacting pathways collectively impair egg production, shell quality, feed efficiency, and hen welfare. The review also synthesizes emerging mitigation strategies across environmental control, nutritional interventions, genetic and breeding innovations, welfare-oriented housing systems, and precision monitoring technologies. Studies indicate that targeted cooling, antioxidant, and electrolyte supplementation, the selection of heat-tolerant strains, enriched environments, and sensor-based early-warning systems can significantly enhance egg-laying hen resilience. Remaining gaps include a limited understanding of multi-stressor interactions, microbiome-mediated thermal tolerance, and the large-scale implementation of precision tools. The review provides a forward-looking framework for improving heat resilience in modern layer systems.

## Simple Summary

Rising global temperatures significantly reduce egg production, eggshell quality, and egg-laying welfare as laying hens are highly sensitive to heat stress. This stress reduces feeding intake, weakens immunity, damages internal organs, and disrupts normal reproductive processes. As a result, heat stress represents both an animal welfare concern and an economic problem for poultry farmers. This review synthesizes recent scientific knowledge on how heat affects the body systems of laying hens with regards to hormone regulation, metabolism, immune function, gut health, and the oxidative balance. It also examines practical strategies that farmers and the poultry industry can use to reduce the negative effects of high temperatures. These strategies include improving housing ventilation and cooling systems, adjusting diets with beneficial nutrients and probiotics, selecting heat-tolerant chicken breeds, improving management and welfare practices, and using new sensor technologies that detect stress early. Understanding how heat stress affects multiple biological systems guides the development of more effective breeding strategies in laying hen production.

## 1. Introduction

The continued increase in global climate change has increased the frequency, duration, and severity of heatwaves, posing a growing threat to poultry-production systems worldwide [1]. Among domestic avian species, laying hens are particularly vulnerable to thermal stress [2]. This is due to their limited capacity for heat dissipation, characterized by the absence of sweat glands and dense feather coverage. Also, high metabolic heat production resulting from the modern genetic selection for egg production contributes to this vulnerability [3,4,5]. Climate projections indicate a continued expansion of regions experiencing hazardous temperature–humidity index (THI) conditions, with direct consequences for layer chicken health, welfare, productivity, and survival [1,6]. In this review, THI values are calculated using the following formula, as described by Kim et al. [7]: THI layers = 0.6 T_db_ + 0.4 T_wb_, where THI = temperature–humidity index (°F), T_db_ = dry-bulb temperature (°F), and T_wb_ = wet-bulb temperature (°F). This index is widely used to assess thermal stress conditions in poultry-production systems. These challenges are more evident in low-income and tropical regions, where poultry production plays a critical role in household nutrition, income generation, and food security [8,9].

Heat stress induces a complex physiological response in laying hens, involving coordinated disruptions in neuroendocrine regulation, metabolism, immune competence, and the redox balance [10]. Exposure to increased temperatures activates the hypothalamic–pituitary–adrenal (HPA) axis, leading to increased circulating corticosterone concentrations and associated alterations in heterophil-to-lymphocyte ratios [11,12]. Under a chronic heat load, sustained HPA axis activation shifts energy metabolism toward maintenance processes at the expense of productive functions, leading to reduced feed intake, impaired nutrient utilization, and diminished egg output [13,14].

Heat stress disrupts acid–base homeostasis through panting-induced respiratory alkalosis, reducing ionized calcium availability for eggshell formation and impairing ovarian and oviductal functions [15,16]. At the same time, an excessive thermal load promotes oxidative stress by increasing reactive oxygen species (ROS) generation, mitochondrial dysfunction, and compromised antioxidant defenses, leading to lipid peroxidation and cellular injury [17,18]. Heat-induced increases in intestinal permeability and gut microbiota dysbiosis further impair immune functions and systemic inflammation. Collectively, these processes contribute to declines in egg mass, shell quality, feed efficiency, and survivability under high-THI conditions [19,20,21].

It is important to note that thermal stress rarely occurs in isolation within commercial production systems. Environmental and managerial stressors, such as elevated ammonia concentrations [22], the ventilation type and structure [23], light intensity [24], the high stocking density [25], and noise exposure [26] interact synergistically with heat load to intensify physiological strain. Evidence from intensive housing systems demonstrates that combined exposure to heat, humidity, and aerial pollutants amplifies oxidative damage, inflammatory responses, immune dysregulation, and reproductive dysfunction beyond the effects of individual stressors alone [22,27]. Furthermore, the global transition toward cage-free and enriched housing systems is largely driven by increasing animal welfare concerns and industry commitments to allow hens to express natural behaviors such as perching, nesting, and dust bathing [28]. While these systems improve behavioral opportunities, they also introduce more complex environmental and social dynamics that can increase exposure to stressors such as competition for resources, social interactions, and variable microclimatic conditions [29]. Consequently, understanding the interaction between housing design and heat stress is becoming increasingly important. Studies have shown that elevated temperatures impair feed intake, the feed conversion ratio, egg production, and eggshell quality, such as breaking strength in layers raised under cage-free conditions [30]. Also, enriched housing systems often incorporate structures such as perches that can influence thermoregulation, as perches can facilitate conductive heat loss through the feet and may be modified as cooling devices to improve thermal comfort during periods of high ambient temperature [31]. Therefore, the transition toward alternative housing systems reshapes both the social and thermal stressors experienced by laying hens.

To mitigate these challenges, diverse heat-stress-mitigation strategies have emerged in recent years. Advances in environmental engineering, such as evaporative cooling systems, cooled perches, ventilation control strategies, optimized airflow distribution, and automated climate-control technologies, have demonstrated potential to reduce the thermal load and improve microclimatic uniformity within poultry houses [32,33,34]. Nutritional interventions, antioxidant supplementation (vitamins C and E, selenium) [35], osmolytes (betaine), trace minerals [36], phytogenic additives, and gut-modulating probiotics and synbiotics have shown efficacy in mitigating oxidative stress, preserving intestinal integrity, and stabilizing metabolic responses under heat stress [36,37,38,39,40,41,42,43,44]. Also, long-term genetic approaches targeting heat tolerance, feather traits, skeletal robustness, and eggshell quality represent promising strategies for climate adaptation in layer populations [3,44,45,46,47]. Early evidence of genetic thermotolerance has been demonstrated in chickens carrying the naked neck (Na) gene, which enhances heat dissipation through reduced feather coverage, as well as in indigenous breeds such as the Fayoumi chicken, which exhibits inherent resilience to high-temperature environments [48,49]. In parallel, precision livestock farming technologies such as thermal imaging, sensor-based THI monitoring, and automated behavior analytics are emerging as valuable tools for real-time stress detection and proactive management [50,51,52].

Although most experimental research on poultry heat stress and environmental control systems has been conducted in temperate regions, a substantial proportion of global poultry production occurs in tropical and developing regions where climatic stressors are more pronounced. The global chicken population exceeded 33 billion birds in 2020, with approximately 46% located in Asia, highlighting the substantial contribution of tropical and subtropical regions to global poultry production [53]. Furthermore, global egg production reached approximately 97 million tons in 2023, underscoring the economic importance of the global layer industry [54]. However, poultry production in tropical regions is frequently constrained by high ambient temperatures and limited climate-controlled housing, which negatively affects productivity and increases mortality [55,56]. Such challenges can lead to significant economic losses across the poultry sector, with studies estimating national losses of approximately USD 98.5 million in commercial poultry farms during crisis periods in developing countries [55]. Consequently, the predominance of research conducted in temperate environments may limit the applicability of existing findings to tropical and sub-Saharan poultry production systems.

This review synthesizes current knowledge to demonstrate that heat stress in laying hens represents a multi-system physiological disruption involving neuroendocrine, metabolic, oxidative, immune, and gastrointestinal pathways. Using an integrated stress physiology framework, it evaluates mitigation strategies spanning environmental engineering, nutritional modulation, genetic selection, housing design, and precision monitoring. The aim is to provide a systems-based framework to improve resilience, productivity, and welfare in layer production under climate change. Accordingly, this study employs an integrative narrative review approach to synthesize mechanistic and applied evidence across multiple physiological systems and mitigation strategies relevant to commercial layer production.

## 2. Literature Search Strategy

This study was conducted as an integrative narrative review to synthesize mechanistic and applied evidence on heat stress physiology and mitigation strategies in laying hen. The objective was not to address a single quantitative research question but to integrate knowledge across multiple physiological systems, such as neuroendocrine, metabolic, oxidative, immune, and gastrointestinal responses, and to evaluate mitigation approaches relevant to commercial egg production. The review emphasized conceptual integration, biological plausibility, and applicability to modern layer-production systems. To operationalize this approach, evidence from the identified studies was synthesized using a thematic integration strategy. Findings were grouped according to major physiological systems affected by heat stress in laying hens, including neuroendocrine, metabolic, oxidative, immune, and gastrointestinal responses. Within each thematic category, results from different experimental models and mitigation strategies were compared to identify consistent physiological mechanisms, converging evidence, and practical implications for commercial layer-production systems.

A structured literature search was performed using Scopus, Web of Science Core Collection, PubMed, and ScienceDirect to identify peer-reviewed studies published between January 2015 and February 2026. This timeframe reflects the period of rapid expansion in climate-related heat stress research in poultry production systems. Search terms combined controlled vocabulary and free-text keywords related to heat stress, physiological responses, and mitigation strategies in layer chickens. Boolean operators were applied as follows: (“heat stress” OR “thermal stress” OR “high ambient temperature” OR “high THI”) AND (“layer chickens” OR “laying hens” OR “egg production”) AND (“stress physiology” OR “neuroendocrine” OR “oxidative stress” OR “immune function” OR “gut integrity”) AND (“cooling” OR “nutrition” OR “antioxidants” OR “genetic selection” OR “housing” OR “precision monitoring”).

This review was conducted as an integrative narrative synthesis rather than a formal systematic review; therefore, study inclusion was not exhaustive and may be subject to selection bias. Differences in experimental design, heat stress models, reporting standards, and outcome measurements limited direct comparison among studies. In addition, differences in heat stress experimental models reported in the literature were considered during the synthesis process. Studies were categorized into acute heat stress (short-term exposure to high temperatures), chronic heat stress (prolonged exposure to elevated temperatures over days or weeks), and cyclic heat stress (alternating thermoneutral and high-temperature periods simulating natural daily temperature fluctuations) [7]. These experimental approaches differ in their physiological outcomes and relevance to commercial production systems; therefore, the model type was considered when interpreting reported effects on metabolism, immune responses, oxidative stress, and production performance. Most available research comes from temperate regions and is conducted under controlled experimental conditions, which may limit its applicability to tropical and small-scale production systems. Within this framework, the methodological quality of individual studies was considered qualitatively during the synthesis process, with emphasis placed on peer-reviewed research employing clearly described experimental designs, appropriate statistical analyses, and relevance to commercial poultry production systems.

## 3. Physiological Mechanisms of Heat Stress in Layer Chickens

Heat stress in laying hen reveals a tightly interconnected network of neuroendocrine, metabolic, oxidative, immune, and gastrointestinal disturbances that undermine physiological stability, reproductive function, and welfare [16,57]. Although these responses have been conventionally examined as distinct processes, accumulating evidence from endocrine, genomic, and transcriptomic studies shows that they operate as components of a unified stress-response system. This integrated stress physiology reflects coordinated central and peripheral adaptations to sustained thermal load [58]. It also explains why heat stress produces systemic effects that extend beyond transient elevations in body temperature.

### 3.1. Neuroendocrine and Metabolic Stress Axis

Exposure to elevated ambient temperatures activates the hypothalamic–pituitary–adrenal (HPA) axis [59,60]. This activation triggers the secretion of corticotropin-releasing hormone (CRH) and arginine vasotocin (AVT) from hypothalamic neurons, as reviewed by previous studies [61,62]. Both neuropeptides act synergistically on pituitary corticotropes to stimulate adrenocorticotropic hormone (ACTH) release, which controls subsequent corticosterone secretion [63]. While acute HPA activation represents an adaptive response to heat stress, prolonged heat exposure results in sustained neuroendocrine stimulation and chronically elevated corticosterone concentrations in laying hens [64]. Under acute heat stress, corticosterone levels increase quickly as an immediate stress response [65]. This response increases energy, blood glucose and adjusts body processes to cope with high temperatures in egg-laying birds [66]. However, during long-term or chronic heat stress, birds may exhibit partial physiological adaptation characterized by relatively lower corticosterone concentrations compared with acute stress responses [67]. Nevertheless, persistent dysregulation of the HPA axis has been reported [65]. These changes show how the body moves from an early emergency response to longer-term physiological adjustments to maintain stability during continuous heat exposure or thermal challenge. Neuroanatomical evidence shows the convergence of CRH- and AVT-expressing neurons onto corticotropes co-expressing CRH1 and VT2 receptors. This provides a mechanistic basis for this amplified ACTH response under chronic stress conditions [63]. This mechanism links persistent heat exposure to endocrine dysregulation and downstream metabolic and immune consequences.

Acute heat stress induces the differential expression of key steroidogenic, antioxidant, and neurosecretory proteins, as shown in the adrenal gland [68]. The study further observes extensive histone post-translational modifications, such as increased H3K27 trimethylation in heat-susceptible chickens. These chromatin-level changes are associated with altered glucocorticoid levels due to stress-induced endocrine reprogramming rather than transient hormonal activation. Proteomic analysis of hypothalamic tissue in layer-type Taiwan country chickens identified 118 differentially expressed proteins following acute heat exposure, including the upregulation of glycolytic enzymes and oxidative-stress-responsive proteins involved in carbon metabolism, glycolysis, and gluconeogenesis pathways [69]. These findings suggest increased hypothalamic glucose utilization and redox buffering to sustain thermoregulatory signaling during heat challenge.

Prolonged glucocorticoid elevation modifies inflammatory responses and compromises physiological resilience, especially when heat stress co-occurs with other environmental or management stressors [62]. Concurrent activation of the sympathetic–adrenal–medullary axis further amplifies catecholamine release and energy mobilization [68]. Integrated transcriptomic–metabolomic analyses further reveal that heat stress disrupts lipid homeostasis, glutathione metabolism, nicotinamide metabolism, and calcium signaling pathways in the hypothalamus and pituitary [70]. This validates the central role of neuroendocrine regulation in coordinating systemic stress adaptation.

Acute restraint stress in 14-week-old chickens increases plasma corticosterone, Met-enkephalin, insulin, and glucose, alongside the upregulation of proenkephalin (PENK) expression in pituitary and adrenal tissues. This confirms the activation of an opioid-mediated stress pathway [71]. Pharmacological blockade with naltrexone partially attenuates stress-induced increases in corticosterone, Met-enkephalin, and glucose, while sustained hyperinsulinemia persists. This suggests stress-induced insulin resistance and the presence of an opioid-modulated neuroendocrine–metabolic axis operating alongside classical glucocorticoid signaling. Also, restraint stress in pullets elevates circulating epinephrine, norepinephrine, thyroxine (T_4_), and triiodothyronine (T_3_), reflecting the coordinated activation of adrenal and thyroid axes [72]. In contrast, chronic thermal exposure consistently suppresses circulating T_3_ concentrations across genotypes, reducing the basal metabolic rate and limiting endogenous heat production as an adaptive response to high ambient temperatures [73]. However, this metabolic adjustment is accompanied by reduced productive performance. Thyroid hormones, particularly T_3_, play important roles in regulating energy metabolism, ovarian activity, and reproductive hormone synthesis [74,75]. Reduced T_3_ concentrations may impair follicular development and steroidogenesis by altering metabolic activity within ovarian tissues and disrupting the secretion of luteinizing hormone (LH) and follicle-stimulating hormone (FSH), thereby contributing to declines in egg production [76]. Dietary rapeseed supplementation has also been associated with reduced T3 activity and decreased egg production in laying hens [77].

Both thyroid and parathyroid hormones influence mineral metabolism and calcium homeostasis, which are critical for eggshell formation [78,79]. Decreased T_3_ levels reduce calcium availability for shell calcification, leading to weaker eggshell structure and reduced shell quality [80]. In addition, studies in broiler chickens suggest that the triiodothyronine-to-thyroxine (T_3_/T_4_) ratio may serve as a physiological indicator of metabolic adaptation to heat stress [81]. However, this indicator requires validation in laying hens. A reduced T_3_/T_4_ ratio reflects decreased peripheral conversion of T_4_ to the biologically active T_3_, indicating suppressed metabolic activity and endocrine adjustments aimed at minimizing heat production. Thus, changes in the T_3_/T_4_ ratio may provide a useful diagnostic indicator of heat-stress-induced metabolic suppression associated with reduced productivity in laying hens.

In addition to activating the HPA axis, heat stress can also disrupt the hypothalamic–pituitary–gonadal (HPG) axis, which plays a central role in regulating reproduction and egg production in laying hens [82]. The HPG axis functions through the pulsatile secretion of gonadotropin-releasing hormone (GnRH) from the hypothalamus, which stimulates the anterior pituitary gland to release luteinizing hormone (LH) and follicle-stimulating hormone (FSH) [83]. These gonadotropins subsequently regulate ovarian follicle development, steroid hormone production, and ovulation [84]. Disruption of this regulatory network can directly impair reproductive hormone secretion and laying performance. Studies demonstrate that increased expression of hypothalamic regulatory genes, including adaptor-related protein complex 2 mu 1 subunit (AP2M1), can inhibit GnRH synthesis and secretion, thereby negatively regulating egg production in chickens [85]. Similarly, reproductive regulation within the hypothalamus involves neuroendocrine pathways mediated by neuropeptides such as tachykinin-3 (TAC3) and prodynorphin (PDYN), which modulate GnRH expression through estrogen-responsive signaling mechanisms in the hypothalamus [86]. These findings indicate that environmental stressors, including heat stress, can disrupt hypothalamic neuroendocrine signaling pathways controlling GnRH release, leading to reduced LH and FSH secretion, impaired follicular development, and a decline in egg production in laying hens.

The peripheral administration of Met-enkephalin increases basal catecholamine levels but decreases stress-induced rises in epinephrine, norepinephrine, T_3_, and T_4_. These findings highlight the inhibitory role of opioid peptides in constraining excessive neuroendocrine excitation.

While phoenixin-14 (PNX-14)-induced hyperphagia in neonatal chickens is mediated by NPY1/NPY5 receptors and CRF1/CRF2 signaling [87], heat-stressed laying hens consistently exhibit suppressed feed intake despite increased metabolic demands. The experimental separation of thermal and nutritional effects demonstrates that heat-induced alterations in the cecal microbial composition are driven predominantly by reduced feed intake rather than an elevated core body temperature [88]. Microbial profiles under cyclic heat exposure closely resemble those observed under pair-feeding conditions [89]. This nutritionally mediated microbial shift coincides with declines in egg production and egg quality under high temperature–humidity-index conditions. This feed intake reduction serves as a central mechanistic link among heat stress, gut dysbiosis, and impaired laying performance. Collectively, these findings indicate that thermally induced anorexia not only constrains nutrient availability for egg formation but also reshapes the gut microbial structure, compounding endocrine and metabolic strain in heat-stressed laying hens

Metabolomic evidence shows that corticosterone redirects energy substrates toward gluconeogenesis and muscle catabolism at the expense of anabolic processes, with altered carbohydrate and amino acid metabolism across hepatic, renal, and muscular tissues [90]. Concurrently, heat-induced panting leads to respiratory alkalosis, elevating blood pH and reducing ionized calcium availability. This thus impairs calcite deposition in the shell gland, resulting in thinner, weaker eggshells with increased breakage rates [16,91]. Chronic heat exposure also engages sympathetic–adrenergic signaling and gut–brain–microbiota communication pathways, increasing metabolic demand while predisposing birds to immune dysregulation and inflammatory imbalances [88]. Importantly, stress-axis activity is subject to inhibitory control. Neuropeptide W (NPW), recently identified as a corticotropin-releasing inhibitory factor in chickens, suppresses ACTH synthesis and secretion via its receptor NPBWR2, while progesterone upregulates pituitary NPBWR2 expression through genomic and non-genomic mechanisms, linking reproductive hormones directly to stress-axis modulation.

These findings demonstrate that neuroendocrine and metabolic responses to heat stress extend beyond corticosterone hypersecretion and instead reflect the coordinated dysregulation of CRH–AVT signaling, opioid peptides, catecholamines, thyroid hormones, appetite-control circuits, insulin-glucose metabolism, and the respiratory acid–base balance. Inter-individual variation in immune–neuroendocrine phenotypes further shapes stress-coping capacity, determining the severity of physiological instability that ultimately compromises reproductive performance, health, and welfare in heat-stressed laying hens.

### 3.2. Oxidative Stress and Mitochondrial Dysfunction

Heat stress imposes a substantial oxidative burden on laying hens by disrupting redox homeostasis and affecting endogenous antioxidant defense systems [92]. This impacts organs essential for egg production. Laying hens function close to their redox threshold due to sustained ovarian activity and hepatic lipogenesis, making them vulnerable to thermally induced oxidative stress [93]. Chronic heat exposure reduces total antioxidant capacity and suppresses key enzymatic defenses (superoxide dismutase, glutathione peroxidase, and catalase), allowing reactive oxygen species (ROS) to accumulate and cause cumulative cellular injury [94].

Mitochondria are recognized as the primary targets and amplifiers of heat-induced oxidative damage in laying hens [95]. Mitochondrial ROS are mainly generated during oxidative phosphorylation within the electron transport chain [96]. The electrons derived from NADH and FADH_2_ are transferred sequentially through respiratory complexes I–IV to generate ATP through complex V [97]. During this process, a fraction of electrons may escape from the respiratory chain and react with molecular oxygen to form superoxide radicals [98]. Electron leakage occurs predominantly at respiratory complexes I (NADH: ubiquinone oxidoreductase) and III (cytochrome bc1 complex) [98]. Complex I typically release superoxide into the mitochondrial matrix during electron transfer from NADH to coenzyme Q, whereas complex III can generate ROS toward both the mitochondrial matrix and intermembrane space through the Q-cycle mechanism [98]. Disruptions in electron transport efficiency increase the electron leakage and amplify mitochondrial ROS generation in poultry tissues. In laying hens exposed to chronic heat stress (33 °C for 4 weeks), renal tissue exhibits reduced ATP levels and mitochondrial DNA copy number [99]. This results from impaired oxidative phosphorylation and compromised mitochondrial biogenesis. Damaged mitochondria leak mitochondrial DNA into the cytosol, activating the cGAS–STING signaling cascade and downstream inflammatory pathways involving MDA5, IRF7, MAVS, and NF-κB, which activate renal inflammation and fibrosis [100]. These findings provide direct evidence that heat stress induces mitochondrial dysfunction and mitochondria-mediated inflammation in laying hens.

The liver is the central hub of lipid synthesis for yolk formation and a critical site of oxidative responses in the chicken. Studies in laying hens have observed that hepatocellular oxidative stress is accompanied by mitochondrial structural damage, cytochrome c release, caspase activation, and the upregulation of ER-stress markers such as GRP78 and CHOP, leading to fibrosis, bile duct hyperplasia, and inflammatory infiltration in the liver [101]. These alterations directly compromise lipid export and yolk precursor synthesis, linking mitochondrial dysfunction to reduced reproductive efficiency. Heat-associated oxidative stress further aligns with fatty liver hemorrhagic syndrome in laying hens. The disruption of mitochondrial energy sensing and amino acid signaling pathways alters lipid synthesis and utilization, impairing oxidative stress and inflammatory responses [102]. In layers, an impaired mitochondrial redox balance suppresses fatty acid oxidation while enhancing lipogenesis, accelerating hepatic lipid deposition and metabolic instability.

Hydrogen-peroxide-induced oxidative stress in laying hens causes mitochondrial structural damage, increased hepatic reactive oxygen species production, activation of inflammatory signaling pathways (NF-κB, TNF-α, IL-1β), and significant lipid accumulation, together with reduced egg production, egg quality, and antioxidant enzyme activities [103]. Oxidative challenge also alters the expression of mitochondrial-associated proteins and genes involved in mitochondrial-endoplasmic reticulum signaling and lipid metabolism [104]. This indicates impaired mitochondrial function and disrupted hepatic energy regulation. Dietary supplementation with chlorogenic acid mitigates these effects by improving antioxidant capacity, preserving mitochondrial integrity, reducing hepatic inflammation and lipid accumulation, and restoring laying performance and egg quality, with 800 mg/kg identified as the most effective dose [103]. These findings demonstrate that mitochondrial oxidative dysfunction represents a key mechanistic link among oxidative stress, hepatic metabolic disturbance, inflammation, and productivity loss in laying hens exposed to environmental stressors. Moreover, mitochondrial oxidative imbalance can impair oxidative phosphorylation efficiency and further increase ROS leakage, creating a self-reinforcing cycle of mitochondrial dysfunction and oxidative injury in poultry tissues [105]. Mitochondrial oxidative dysfunction acts as a central convergence point through which neuroendocrine activation, metabolic strain, immune signaling, and reproductive demand are translated into systemic pathology under heat stress.

### 3.3. Immune and Inflammatory Dysregulation

Long-term exposure to high temperature with elevated ammonia concentrations suppresses mucosal immunity, as indicated by reduced IgA levels, and increased circulating IgG reflecting an immune imbalance rather than enhanced protection [106]. These immune alterations correspond to reduced total antioxidant capacity and the suppression of reproductive hormones (LH, FSH, and estradiol), linking inflammatory stress directly to impaired ovarian function. In the same study, corticosterone responses were transient, whereas immune and endocrine disruptions persisted into mid and late lay. This indicates that chronic environmental stressors drive sustained immune–reproductive dysregulation independent of acute HPA activation.

Comparative studies demonstrate that reductions in feed intake, egg production, egg quality, and immune competence vary markedly among genotypes, with Golden Sabahi hens exhibiting greater thermotolerance and smaller declines in shell quality and production traits than Lohmann Brown, Fayoumi, or White Leghorn strains [107]. These differences are mirrored by strain-specific alterations in organ development, including liver hypertrophy, pancreatic enlargement, abdominal fat deposition, and differential ovarian follicle dynamics under acute heat stress, underscoring the tight coupling among immune stress, metabolic reprogramming, and reproductive capacity.

Transcriptomic analyses reveal that heat stress alters antigen presentation and innate immune responsiveness through the differential regulation of stress-responsive genes such as DUSP1 and HSPA5, which modulate MHC-II expression, costimulatory signaling, and sensitivity to inflammatory stimuli [108]. These findings provide mechanistic support for the observed decline in vaccine responsiveness and increased disease susceptibility under heat stress. Importantly, nutritional modulation can partially restore immune competence: supplementation with curcumin enhances antioxidant enzyme activity, elevates IgG and IgA concentrations, improves reproductive hormone profiles, and restores laying performance under heat stress, confirming that immune dysfunction is mechanistically linked to oxidative and endocrine stress rather than representing an inevitable consequence of elevated temperature [109]. These studies demonstrate that immune dysregulation in heat-stressed laying hens involves the interaction of thermal load, oxidative stress, endocrine disruption, and intestinal barrier dysfunction. Suppressed protective immunity coupled with persistent inflammatory activation compromises disease resistance, reproductive efficiency, and welfare.

The coexistence of immunosuppression and systemic inflammation under heat stress reflects differential regulation of innate and adaptive immune responses. Heat stress increases oxidative stress and intestinal permeability, which promotes endotoxin translocation and the activation of inflammatory signaling pathways such as NF-κB. This process stimulates the production of pro-inflammatory cytokines, including IL-1β, IL-6, and tumor necrosis factor-α, resulting in systemic inflammatory responses in heat-stressed laying hens [110].

At the same time, chronic heat exposure elevates corticosterone levels and disrupts endocrine and metabolic homeostasis, which suppresses adaptive immune functions such as lymphocyte proliferation and antibody production. Experimental evidence shows that heat stress reduces B cells, helper T cells, and circulating cytokines associated with adaptive immunity, including IL-2 and interferon-γ, indicating impaired humoral and cellular immune responses [111]. This imbalance between pro-inflammatory signaling and reduced adaptive immune competence is further supported by studies showing that heat stress increases oxidative damage and inflammatory gene expression while reducing antioxidant capacity and immune function in the liver and other tissues [112]

Moreover, heat stress can impair reproductive physiology through inflammatory and apoptotic pathways. Activation of the FasL/Fas and TNF-α signaling systems in ovarian follicles induces the apoptosis of follicular cells, reduces hierarchical follicle numbers, and ultimately decreases egg production. These findings highlight how immune-inflammatory responses under heat stress extend beyond host defense mechanisms and contribute directly to reproductive and metabolic dysfunction.

Together, these observations indicate that heat stress induces a paradoxical immune phenotype characterized by the overactivation of innate inflammatory pathways alongside the suppression of adaptive immune responses, which ultimately increases disease susceptibility, reduces productivity, and compromises welfare in laying hens.

### 3.4. Gastrointestinal Permeability and Microbiome Alteration

The gastrointestinal tract represents one of the most heat-sensitive physiological systems in laying hens under different dietary conditions [113,114,115]. Also, it serves as an interface through which thermal stress propagates systemic metabolic and immunological dysfunction [116]. Exposure to elevated temperatures rapidly compromises intestinal barrier integrity through the downregulation of tight-junction proteins (zonula occludens-1, occludin, and claudins), resulting in increased paracellular permeability. Structural alterations to the intestinal mucosa, such as a reduced villus height, increased crypt depth, and impaired antioxidant capacity across the duodenum, jejunum, and ileum, have been consistently observed under chronic heat load in laying hens [117]. Another study investigated the influence of chlorogenic acid (CGA) on gut morphology and cecal microbiota in late-peak laying hens under heat stress. The dietary inclusion of 600 and 800 mg/kg CGA significantly upregulated the duodenal, jejunal, and ileal ZO-1 and occludin gene expression and changed the gut microbiota structure by increasing short-chain fatty acid (SCFA)-producing bacteria and *Lactobacillus*, *Bacillus*, and *Akkermansia* [118]. These alterations reduce the absorptive surface area, accelerate epithelial turnover, and predispose birds to the translocation of luminal antigens, endotoxins, and microbial metabolites into systemic circulation.

In laying hens, thermal challenge consistently reshapes the gut microbial community, typically reducing the abundance of *Firmicutes*—particularly SCFA-producing taxa and increasing the proportions of *Bacteroidetes* and *Proteobacteria,* a microbial configuration associated with inflammatory and metabolic instability [119]. Functional predictions and metabolomic analyses in broilers indicate that the disruption of microbial pathways involved in amino acid metabolism, lipid processing, retinol metabolism, and xenobiotic degradation, linking microbial imbalances to hepatic stress and impaired nutrient utilization [120]. These compositional and functional shifts reduce fermentative efficiency and SCFA availability to enterocytes, thereby weakening epithelial energy supply and reinforcing barrier dysfunction.

Microbiota alterations under heat stress may arise from both direct thermal effects on intestinal physiology and secondary changes associated with reduced feed intake [121,122]. Heat exposure itself can damage the intestinal epithelial structure, disrupt mucosal immunity [123], and increase circulating corticosterone levels [124]. This thereby creates an intestinal environment that favors microbial dysbiosis. The exposure of laying hens to high ambient temperatures (34 °C) reduces feed intake and egg production while simultaneously causing villus damage, inflammatory cytokine release, and reduced mucosal immune cell populations, indicating that thermal stress can directly impair intestinal homeostasis independent of nutritional restriction [114].

In pullets subjected to daily thermal oscillation, cecal microbial diversity and total bacterial abundance decline markedly even under moderate heat conditions, with significant reductions in *Firmicutes* observed across both gastrointestinal and respiratory areas [113]. These microbiome changes coincide with decreased feed intake, altered heterophil-to-lymphocyte ratios, and imbalances in the CD4^+^/CD8^+^ T cell population in laying hens [125]. This underscore tight functional coupling among the thermal environment, gut microbiota, and systemic immune status during early layer development. Importantly, these findings demonstrate that fluctuating thermal stress, rather than sustained hyperthermia alone, is sufficient to destabilize microbial homeostasis and suppress beneficial taxa.

However, reduced feed intake remains an important confounding factor when interpreting microbiome changes during heat stress. Feed restriction alters nutrient flow to the lower gastrointestinal tract and can independently reshape microbial communities by modifying substrate availability for fermentation. Consequently, experimental designs that incorporate pair-fed controls or nutritional adjustment strategies are necessary to disentangle the direct thermal effects of heat stress from secondary dietary influences on microbial composition [126]. Evidence from probiotic intervention studies further supports a causal role of microbial regulation in performance responses during heat stress [127,128,129]. Supplementation with a probiotic mixture containing *Bacillus subtilis* and *Enterococcus faecium* improved egg production, feed intake, eggshell quality, and intestinal barrier integrity in heat-stressed laying hens [122]. This explains the association of improved gut microbial balance and reduced bacterial invasion of intestinal tissues.

Dysbiosis under thermal load is associated with the elevated expression of heat-shock proteins such as HSP70 within intestinal tissues, reflecting cellular stress responses to oxidative and inflammatory challenge in broiler chickens [130]. Further research is needed to determine whether similar responses occur in laying hens. Concurrent shifts in circulating and intestinal metabolomic profiles indicate altered energy partitioning, intensified proteolysis, and redox imbalances, reinforcing the role of the gut as both a target and amplifier of systemic stress [131]. Increased intestinal permeability facilitates endotoxin leakage, promoting low-grade systemic inflammation and placing additional metabolic demands on the liver and immune system.

Heat stress disrupts hepatic lipid metabolism, which contributes to metabolic imbalances in poultry [132]. Thermal stress promotes hepatic lipid accumulation through the transcriptional reprogramming of lipid metabolic pathways [132,133]. Specifically, heat stress can upregulate sterol regulatory element-binding protein-1c (SREBP-1c), a transcription factor that stimulates de novo lipogenesis, while simultaneously suppressing peroxisome proliferator-activated receptor-α (PPARα), which normally promotes fatty acid β-oxidation [134]. The downregulation of PPARα under heat stress has been associated with increased inflammatory responses and impaired metabolic homeostasis in poultry tissues.

Oxidative stress induced by heat exposure can activate endoplasmic reticulum stress (ERS) pathways [135]. Glucose-regulated protein 78 (GRP78) serves as a key sensor of ER stress, while prolonged ER stress activates the transcription factor CCAAT/enhancer-binding protein homologous protein (CHOP), which is associated with cellular dysfunction and apoptosis [136]. Heat-stressed hens show elevated oxidative stress and ER stress markers are accompanied by reduced antioxidant enzyme activity and disruption of reproductive hormone regulation, highlighting the systemic metabolic impact of hyperthermia [137]. These findings indicate that ER stress and the transcriptional dysregulation of lipid metabolism jointly contribute to hepatic lipid accumulation and metabolic instability during heat stress. Supplementation with glycerol monolaurate (325 mg/kg) improved egg production, egg weight, eggshell strength, and Haugh units while restoring intestinal barrier integrity in heat-stressed laying hens [138]. Also, GML alleviated hepatic steatosis by reducing lipid droplet accumulation, plasma lipoprotein cholesterol (LDL-C), aminotransferase (AST), and alanine aminotransferase (ALT), and hepatic triglyceride levels while increasing high-density lipoprotein cholesterol (HDL-C). The study further revealed the activation of fatty acid β-oxidation and sphingolipid metabolism pathways, with ACSL1 and CPT1A identified as key regulatory genes associated with improved production performance and gut–liver health. These findings suggest that the modulation of hepatic lipid metabolism and enhancement of fatty acid degradation may represent effective nutritional strategies to improve laying hens’ resilience and productivity under heat stress [139].

Despite the dominance of broiler-based gastrointestinal heat-stress research, available evidence suggests that laying hens may be particularly vulnerable to chronic gut dysfunction due to their prolonged production cycles, sustained metabolic heat output, and continuous nutrient demands associated with egg formation. The disruption of intestinal integrity and microbial homeostasis in layers could have direct and cumulative consequences for egg production, shell quality, nutrient efficiency, and susceptibility to enteric disease, translating into substantial economic and welfare costs at the industry level.

### 3.5. Integrated Perspective: Heat Stress as a Multi-Systemic Biological Integration Problem

**Figure 1:**
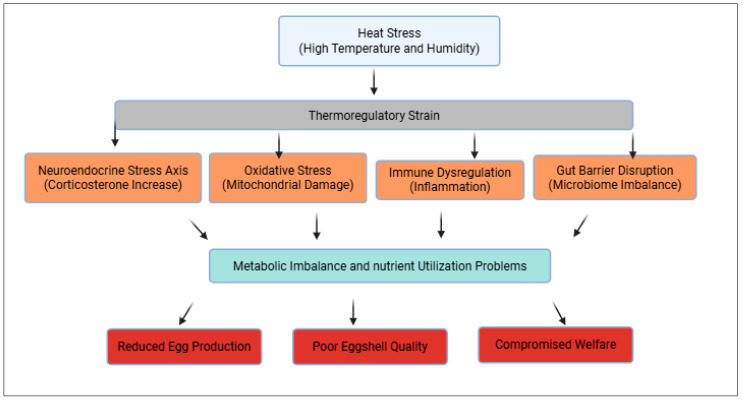
Integrated framework for heat stress in Laying hens.

Research across neuroendocrine, metabolic, oxidative, immune, and gastrointestinal pathways demonstrates that heat stress in layer chickens represents a multi-system biological disorder rather than a localized effect of thermoregulation [136]. Activation of the HPAI endocrine and metabolic reallocation, while panting-induced acid–base disruption compromises calcium homeostasis and eggshell formation [140]. These disturbances spread through mitochondrial dysfunction, amplifying oxidative damage and bioenergetic failure, which in turn suppresses adaptive immunity and promotes maladaptive inflammatory signaling. The concurrent breakdown of intestinal barrier integrity and microbiome homeostasis further reinforces systemic inflammation, endotoxemia, and nutrient inefficiency. Importantly, these pathways do not operate independently but form a self-reinforcing physiological network in which dysfunction in one system amplifies instability in others, ultimately leading to impaired egg production, reduced egg quality, compromised welfare, and increased mortality risk. Recognizing heat stress as an integrated biological syndrome underscores the limitations of single-axis interventions and provides the conceptual foundation for holistic, multi-modal mitigation strategies discussed in the subsequent section (Figure 1).

## 4. Mitigating Factors for Heat Stress in Layer Chickens

Given the increasing impact of climate change, it is essential to assess the resilience of laying hen breeds to environmental stressors. In addition, developing new genetic lines, particularly those with enhanced heat tolerance, will be critical to meet the production demands of farmers and the quality expectations of consumers. The effective management of environmental factors remains a cornerstone of successful poultry production and welfare [141]. Such management factors entail improving housing ventilation [142], installing cooling systems [143], and adjusting dietary formulations to reduce metabolic heat production and maintain the electrolyte balance during high-temperature conditions [144]. Supplementation with vitamins and antioxidants has also been recommended to support birds’ physiological resilience under stress [145,146].

### 4.1. Housing Systems

Housing systems fundamentally shape the thermal, social, and behavioral conditions experienced by laying hens, and are central to welfare outcomes. Under heat stress, the interaction among the structural design, ventilation efficiency, and stocking density influences hens’ ability to dissipate metabolic heat and perform the adaptive behaviors necessary for thermoregulation [147]. Once temperatures exceed the thermoneutral zone, hens rapidly exhibit physiological strain, behavioral restriction, and reduced health and productivity [148]. Housing structures vary in their capacity to buffer birds from environmental heat. Open-sided houses typical of tropical regions allow natural ventilation yet provide limited protection against rising temperature and humidity [149,150]. These conditions reduce evaporative heat loss, leading to panting, wing-elevation postures, and peripheral vasodilation, behavioral and physiological indicators of thermal discomfort [150,151]. In contrast, climate-controlled systems with insulated roofing, optimized airflow pathways, and engineered ventilation maintain more stable indoor climates [152]. Air velocities of 2–3 m/s substantially improve convective heat loss and are beneficial for older or high-producing hens with greater metabolic heat output [150,153]. Such thermal cushioning directly enhances welfare by mitigating the physiological heat load and reducing reliance on energetically costly panting.

However, the economic feasibility of advanced housing technologies remains an important consideration for producers, particularly in resource-limited settings [154]. Mechanical ventilation and evaporative cooling systems typically require continuous electrical input for fans, pumps, and control systems, which can increase farm energy consumption [155]. Energy requirements for poultry environmental control systems have been estimated to represent a significant proportion of total farm operational costs, emphasizing the importance of energy-efficient design and insulation in poultry housing [156].

High densities hinder airflow at the bird level, elevate local humidity, and intensify heat accumulation, which further explains the impact of stocking density on layers’ welfare [157]. These conditions exacerbate heat stress, suppress feed intake and body weight gain, and impair immune function. Overcrowding limits hens’ ability to disperse, avoid dominant flock mates, or position themselves in cooler microhabitats, increasing social tension and feather pecking while elevating corticosterone levels [158,159]. Thus, density-driven constraints simultaneously worsen thermal, social, and physiological welfare. Also, hens increase water consumption substantially during heat stress to compensate for evaporative losses, making drinker availability and distribution essential [151]. Inadequate access to cool, clean water heightens the dehydration risk and exacerbates performance declines. In another situation, insufficient ventilation permits the accumulation of ammonia and other noxious gases, contributing to respiratory irritation, mucosal damage, and increased susceptibility to disease [160]. Analytical approaches integrating environmental data and production records further demonstrate the critical role of housing microclimate management. Machine-learning models developed using large commercial datasets have shown that the environmental temperature, air speed, and flock age strongly influence egg production performance, enabling predictive decision-support tools for ventilation or cooling interventions under hot weather conditions [161]. Climate-control strategies, including insulation, mechanical ventilation, and evaporative cooling, are therefore central to preventing the cascade of heat-induced reductions in feed intake, egg production, and immune competence. Beyond thermal regulation, housing systems influence behavioral welfare. Comparative evidence indicates that enriched colony cages and aviary systems support greater behavioral diversity and reduce indicators of stress when contrasted with conventional battery cages [162]. Therefore, housing systems promote welfare most effectively when they integrate effective thermal buffering through insulation, ventilation, and airflow, adequate space and enrichment to enable behavioral thermoregulation and reduce social stress and reliable access to air and water resources that meet hens’ elevated physiological demands during heat.

### 4.2. Nutrition

Heat stress (HS) imposes a synergistic burden on laying hens by disrupting the energy balance, acid–base homeostasis, gut integrity, and oxidative stability, which reduces egg production and compromises welfare [68]. Nutritional strategies represent a practical and scalable mitigation approach through biochemical, metabolic, and physiological pathways. Across various studies, there have been interventions with betaine, antioxidants, trace minerals, probiotics, and multivitamin combinations (Table 1). Studies have shown a consistent pattern in single nutrients alleviating selective aspects of HS physiology, whereas broader combinations show potential to target multiple biological systems simultaneously.

Trials conducted in commercial hens under elevated THI conditions demonstrate that betaine improves the metabolic status and preserves key components of laying [163]. Further research shows that higher inclusion levels (3–6 g/kg feed) modulate intestinal tight-junction gene expression, enhancing epithelial barrier function during hot seasons [164]. When used to replace methionine partially, betaine improves egg production and serum lipid profiles while interacting positively with supplemental zinc [165]. Across studies, the mechanistic actions of betaine align with improved welfare indices, including reduced hepatic stress enzymes, enhanced serum protein synthesis, and protection against dehydration-driven osmotic instability.

Vitamin C plays a key role in maintaining adrenal function and reducing corticosteroid overproduction during thermal load. It has been shown to counteract HS-induced hepatic lipid accumulation and normalize metabolic pathways associated with energy and amino acid metabolism [166]. Vitamin E (lipid-soluble antioxidant) and Vitamin C when combined with selenium, yields synergistic oxidative protection, reflected in improved feed efficiency, normalized hematological stress markers, and enhanced productive performance under high temperatures [167]. These findings collectively underscore that antioxidant supplementation not only mitigates oxidative injury but also contributes to maintaining physiological homeostasis that supports welfare and productivity under HS.

Trace mineral supplementation, such as zinc and chromium, plays a central role in maintaining laying performance and metabolic stability under environmental stress. Evidence from controlled factorial feeding trials using aged Lohmann-LSL Lite hens (65 weeks old) fed corn–soybean diets (2750 kcal/kg ME; 14.69% CP) demonstrated that zinc sulfate supplementation at 40 mg/kg across replicated cage treatments over 12 weeks significantly increased egg weight and egg mass and improved the feed conversion ratio [168]. In another study, chromium supplementation with Lohmann lite hens exposed to heat-stress conditions (34 ± 2 °C for 8 h daily over 12 weeks) showed that dietary chromium supplied as chromium-picolinate or chromium-histidinate (200 μg) partially alleviated heat-stress-induced declines in feed intake, egg production, egg weight, and metabolic disturbances such as elevated serum glucose and cholesterol, although egg quality traits were less responsive [169]. In related trials using the same environmental model, chromium supplementation improved nutrient digestibility and upregulated intestinal glucose, amino-acid, and fatty-acid transporter expression, indicating enhanced nutrient utilization under heat stress, with chromium-histidinate showing slightly greater bioavailability [170]. Earlier feeding studies in Babcock layers comparing inorganic, organic, and nano-chromium sources at 200–400 μg/kg diets for 12 weeks reported improved mineral retention and tissue mineral accumulation without a notable effect on egg production (). This explains chromium’s role in mineral metabolism [171]. Collectively, these findings show that zinc primarily supports antioxidant defense and shell mineralization, whereas chromium improves glucose homeostasis, nutrient utilization, and mineral metabolism; together, these trace minerals contribute to maintaining metabolic stability and production efficiency in laying hens exposed to heat or other environmental stressors.

Growing restrictions on antibiotic growth promoters have increased interest in probiotics and related microbiome-targeted strategies to improve laying hen resilience under environmental stress. Lohmann pink laying hens’ supplementation with *Clostridium butyricum* (0.5 g/kg feed) improved feed conversion, eggshell strength, and the albumen protein content. Also, probiotic combinations of *Saccharomyces boulardii* and *Pediococcus acidilactici* reduced reactive oxygen species in intestinal tissues and serum malondialdehyde under heat stress, indicating improved gut health and oxidative status [172]. Under high ambient temperature, three chicken laying breeds fed diets containing 0, 200, or 400 ppm probiotics for 90 days reported improved shell thickness, reduced plasma cholesterol and triglycerides, and increased IgM concentrations without adverse effects on production traits, demonstrating enhanced immune competence and egg quality during heat challenge [129]. Similarly, trials with Isa White layers exposed to cyclic heat stress for five months showed that dietary *Lactobacillus plantarum* RS5 improved egg production, egg weight, and Haugh units compared with non-supplemented controls [173]. This suggests that microbiome-derived metabolites may alleviate heat-induced productivity losses.

However, the functional effects of probiotics vary considerably among microbial genera due to differences in metabolic characteristics, ecological niches, and mechanisms of action [43]. Lactobacilli are primarily lactic acid-producing bacteria that colonize the intestinal mucosal surface and contribute to host health by lowering the intestinal pH, producing antimicrobial compounds, and enhancing intestinal barrier function. Supplementation with *Lactobacillus rhamnosus* has been shown to improve eggshell quality and lipid metabolism in laying hens, partly through the modulation of intestinal microbiota and nutrient utilization [174]. In contrast, Bacillus species are spore-forming bacteria that survive feed processing and harsh gastrointestinal conditions [128]. Their primary mode of action involves the secretion of extracellular enzymes (proteases, amylases, and lipases) that enhance nutrient digestion and availability in the intestinal lumen. *Bacillus amyloliquefaciens* (0.01–0.06% of diet) significantly increases egg production and egg mass and enhances eggshell strength and thickness of layer chicken under heat stress [127]. In another study, dietary Bacillus subtilis at 200–400 g/t feed improved eggshell thickness, eggshell breaking strength, and egg quality under high-ambient-temperature conditions [175]. Yeast-based probiotics differ substantially from bacterial probiotics as they function mainly through metabolic and immunomodulatory pathways rather than intestinal colonization [176]. Yeast cells provide bioactive compounds such as β-glucans, mannan oligosaccharides, and antioxidant micronutrients that enhance immune responses, improve antioxidant capacity, and modify the gut microbial composition [176,177,178]. Dietary supplementation with selenium-enriched *Saccharomyces cerevisiae* has been reported to improve egg production in laying hens exposed to heat stress [179]. Collectively, these findings indicate that probiotic supplementation can improve productivity and physiological resilience in laying hens under heat stress through multiple mechanisms (Table 1). However, the effectiveness of probiotic interventions depends strongly on the microbial species and strain used, emphasizing the need for the targeted selection of probiotic genera with complementary functional properties.

Multivitamin blends combining vitamins A, C, E, K, folic acid, and selenium demonstrate multi-target effects, enhancing feed intake, antioxidant activity, and egg quality attributes in heat-stressed layers [145]. These combinations outperform single vitamins in many cases, likely because they simultaneously target adrenal stress, oxidative pathways, and membrane integrity. However, their complexity introduces challenges in mechanistic attribution and dose standardization.

Although nutritional strategies consistently affect at least one of the physiological burdens imposed by HS, the heterogeneity in study designs, nutrient doses, bird genotypes, and HS intensity complicates direct comparisons. A clear pattern emerges that nutrients that target oxidative damage, osmotic stress, and gut integrity reliably improve welfare indicators, while improvements in productivity (egg production, egg mass, shell quality) are more variable and often incomplete (Table 1). This suggests that nutritional strategies for mitigation do not fully counteract the biological disruption caused by thermal stress.

Despite substantial progress, major knowledge gaps remain. Dose–response relationships for most nutrients, particularly in multi-component interventions, are poorly defined. Interactions between environmental control strategies and nutritional supplementation require systematic evaluation. Mechanistic pathways linking nutrition to shell gland physiology, heat-shock signaling, and neuroendocrine modulation are insufficiently characterized. Long-term, field-based studies incorporating welfare metrics—feather condition, behavior, and immune competence—are notably absent. Future work should prioritize factorial designs testing nutrition × environment × genotype interactions, supported by omics-based biomarker discovery to enable precision nutrition under increasing climatic variability.

**Table 1:** Nutritional interventions for heat stress mitigation in laying hens: Effects on physiology, welfare, and performance.

References	Age of Hens	Nutrient, Dosage, and Concentration	Sample Size	Duration	Effects on Welfare/Physiology	Effects on Productivity
[180,181]	22 weeks	Betaine at 0, 200, 400, 600 mg/kg under THI > 72	600 hens; 5 groups; 8 replicates × 15 hens	14 weeks	Heat stress increased GOT, GPT, CK; reduced TP and AKP; betaine lowered CK/GPT; improved protein metabolism	400–600 mg/kg increased hen-housed egg yield and laying rate
[164]	32 weeks	Betaine 3 g/kg or 6 g/kg	216 hens (3 diets × 6 replicates × 12 hens)	6 weeks	Increased occludin and claudin-1; reduced ZO-1 and JAM-2 modulated tight-junction gene expression under heat	Trend for increased hen-day production; reduced broken/shell-less eggs; stronger shells
[166]	12 weeks (growing pullets)	Vitamin C 300 mg/kg	Not specified	3 weeks	Vitamin C reduced hepatic triglycerides, lipid accumulation, and heat-induced metabolic dysregulation	Not production-stage hens; welfare benefit through liver protection
[182]	32–48 weeks	Vitamin E 0, 250, 500 mg/kg; Selenium 0, 0.25, 0.50 mg/kg	288 hens + 36 cocks	16 weeks	Reduced heterophils and eosinophils; increased monocytes; improved antioxidant status	Vitamin E + Se improved feed intake, FCR; highest performance at 500 mg Vit E + 0.50 mg Se
[169]	16 weeks	Chromium picolinate or histidinate delivering 200 µg Cr/kg diet	1800 hens	12 weeks	Lowered serum glucose and cholesterol; replenished serum Cr; partially improved electrolyte balance	Improved feed intake, production, and egg weight; but no improvement in egg quality deterioration
[183]	Not stated	CHM 3.32 g/kg; ginger 10 g/kg; mix CHM + ginger	250 hens	Duration unspecified	Improved antioxidants (CAT, GSH-PX, NO, T-AOC); stabilized serum lipids	Improved egg production, feed intake; H3 (mixed herbs) outperformed all groups
[184]	40 weeks	Fennel 0, 10, 20 g/kg	120 hens	-	Reduced oxidative damage (lower egg MDA and carbonyl); lower yolk triglycerides & cholesterol	Increased egg quality (shell thickness, strength, albumen height); lower broken eggs
[185]	18–25 weeks	AE, KC, CE, ESE, CAF; varying vitamin combinations	28 hens (7 groups × 4 replicates)	7 weeks	Improved antioxidant enzymes (SOD, GPx); reduced MDA; improved immune-oxidative resilience	CE, ESE, and CAF improved eggshell thickness, shape index, HU, and albumen index
[186]	55 weeks	Oregano oil 25–100 mg/kg	300 hens; 5 groups	100 days	Increased villus height and V/C ratio; altered cecal microbiota (Megamonas, Bacteroidales)	Increased eggshell thickness and weight; improved yolk PUFA, riboflavin and thiamine levels
[187]	80 weeks	*Pediococcus acidilactici* CNCM I-4622 (50–200 mg/kg)	180 hens; 5 groups	-	Reduced diamine oxidase and IL-8; increased villus height; reduced crypt depth; down-regulated IFN-γ and TNF-α; improved tight-junction gene expression	No significant change in production performance

THI, temperature–humidity index; GOT, glutamic oxaloacetic transaminase (AST); GPT, glutamic pyruvic transaminase (ALT); CK, creatine kinase; TP, total protein; AKP, alkaline phosphatase; AST, aspartate aminotransferase; CAT, catalase; GSH-PX, glutathione peroxidase; NO, nitric oxide; T-AOC, total antioxidant capacity; MDA, malondialdehyde; SOD, superoxide dismutase; GPx, glutathione peroxidase; FCR, feed conversion ratio; HU, Haugh unit; PUFA, polyunsaturated fatty acids; V/C ratio, villus height to crypt depth ratio; IL-8, interleukin-8; IFN-γ, interferon-gamma; TNF-α, tumor necrosis factor-alpha; CHM, Chinese herbal mixture; AE, KC, CE, ESE, CAF, vitamin combinations.

### 4.3. Precision Farming

Precision-farming technologies demonstrate strong potential for the early detection and mitigation of heat stress in laying hens, yet their field-readiness remains uneven. Thermal-signature modeling represents a notable advance, as Soris et al. [188] showed that face and wattle provide highly discriminative surface-temperature patterns, achieving up to 89% accuracy in understanding heat-stress states of layer chickens. However, this study was conducted under controlled experimental conditions using a relatively small dataset of birds housed in laboratory-scale environments, which may limit the generalizability of the algorithm to large commercial flocks where thousands of birds are present simultaneously.

Similarly, multimodal AI frameworks expand the detection window by integrating environmental, behavioral, and physiological signals. Hayes et al. [189] demonstrated that Convolutional Neural Networks–long short-term memory fusion not only improves predictive accuracy (94.7%) but also identifies deviations before overt clinical stress becomes visible. This temporal modeling capacity is a significant advancement as it takes precision farming from reactive monitoring towards proactive risk prediction. Nevertheless, most of these models were trained and validated using datasets collected from small experimental groups of birds, often fewer than 200 individuals, which differs substantially from commercial layer houses that typically contain 10,000–50,000 birds.

Further, the acoustic classification of vocal stress signatures [190] introduces new welfare biomarkers capable of functioning even under visual occlusion, another notable contribution to the field. However, acoustic models also face scalability challenges because large commercial facilities contain significant background noise generated by ventilation systems, equipment, and flock vocalization density, which may interfere with signal detection and algorithm accuracy.

Low-cost IoT systems further contribute to heat-stress mitigation by providing the continuous, real-time measurement of environmental drivers. The AMCU developed by Elwakeel [191] achieved a high correlation with certified devices (r > 0.96) at a fraction of the commercial cost, illustrating that scalable sensing is feasible even for smallholder producers. Recent developments further highlight the importance of integrating environmental monitoring with artificial intelligence to improve heat-stress management in commercial poultry houses. IoT-based environmental monitoring systems capable of measuring temperature, humidity, and gas concentrations have been successfully deployed in operational layer houses, providing continuous real-time environmental data that can be integrated with machine-learning models to predict environmental risk factors and improve management decisions [192].

In commercial poultry facilities, environmental variables extend beyond temperature and humidity to include air-quality parameters such as carbon dioxide (CO_2_), methane (CH_4_), ammonia (NH_3_), and hydrogen sulfide (H_2_S), which accumulate due to bird respiration, manure decomposition, and microbial activity. Elevated concentrations of these gases can impair respiration, increase disease susceptibility, and reduce egg production and overall flock productivity [193].

Monitoring studies have further demonstrated that gas concentrations in poultry houses exhibit strong diurnal variation and are influenced by ventilation rates, litter conditions, the stocking density, and the environmental temperature (Table 2). These dynamic conditions create complex environmental interactions that may influence bird behavior, welfare, and productivity while simultaneously introducing noise into sensor data streams used by machine-learning algorithms [193].

Although not a direct heat-stress classifier, such environmental monitoring is fundamental to automated control systems and can prevent thermal overload before it occurs. Additionally, vision-based behavioral monitoring systems by Italiya et al. [194] highlight the value of feeding, drinking, and fine-feature detection as indirect but sensitive indicators of heat-induced behavioral disruption. Recent developments in precision poultry farming confirm that many AI and sensor-based systems remain validated primarily in small-scale trials (Table 2). For example, a sensor-integrated AI automation system tested in a commercial shed involved only 20 birds during a 30-day deployment, although it demonstrated reliable operation across 8640 decision cycles and improved egg production by 14.5% through automated ventilation control [195]. Similarly, computer vision frameworks designed to measure head-region temperature through cross-modal RGB–infrared imaging have been validated using controlled datasets of a few hundred images and small experimental flocks, highlighting the need for further testing under commercial production densities where feather coverage, crowding, and movement patterns introduce additional noise into image-based measurements [196].

Several important research gaps remain in the precision monitoring of heat stress in laying hens. First, many models are developed under controlled or small-scale conditions and may not perform reliably in commercial houses where a high stocking density, variable lighting, dust, and sensor occlusion reduce accuracy. For example, feather coverage can interfere with infrared temperature detection because surface temperature readings differ significantly between feathered and featherless body regions, which may introduce measurement bias in automated thermal monitoring systems [196].

Second, multimodal integration remains inconsistent, with no standardized framework for combining thermal, behavioral, acoustic, and environmental data into a unified decision-support system. Third, most studies focus on detection rather than intervention; few evaluate closed-loop systems that automatically trigger cooling, ventilation, or management responses to reduce welfare loss and production declines. In addition, the sensor ergonomics and long-term welfare impacts of wearable devices require further investigation by Li et al. [197], and economic feasibility analyses are limited, particularly for small- and medium-scale producers.

Recent studies highlight three major innovations in precision heat-stress monitoring. The first is anatomically targeted thermal imaging for fine-scale physiological detection [198], multimodal AI models that incorporate temporal patterns to forecast stress events [199], and low-cost IoT and edge-computing tools that expand adoption across layer’s production [200,201]. Artificial intelligence-driven monitoring systems are increasingly integrated into precision poultry farming platforms that combine sensors, computer vision, and predictive analytics to support real-time flock management and welfare monitoring [202]. However, several gaps remain. Model robustness under commercial conditions is uncertain, given variability in airflow, dust, lighting, and flock density. Methodological inconsistency across studies—particularly in defining thermal regions, behavioral metrics, and environmental thresholds—limits multimodal integration and cross-study comparisons. Moreover, few investigations test closed-loop management systems to determine whether sensor-triggered interventions meaningfully improve welfare or productivity, and the early-life integration of monitoring tools remains understudied despite evidence that rearing conditions shape later thermal resilience.

Overall, precision technologies show strong promise as early warning systems, but future research should prioritize standardized multimodal datasets, large-scale commercial validation, biologically meaningful stress thresholds, and economically viable closed-loop frameworks that not only detect heat stress but also demonstrably reduce its impacts on hens’ welfare and production.

**Table 2:** Sensor-based and AI-driven systems for early detection of heat stress in laying hens.

Reference	System/Sensors	Sample Size and Scale	Key Method (ML/Metric)	Main Performance/Finding	Implication for Heat-Stress Mitigation
[188]	Infrared thermography (face, eye, wattle, comb, leg, foot)	192 layers, climate-controlled chambers	Thermal signature + classifiers (Random Forest best)	RF face: 89.0%; wattle: 88.3%	Region-specific thermography + ML can classify heat risk from surface temp, suitable for automated alerts
[189]	Enviro sensors (T, RH), thermal imaging, motion	Layer houses, 90-day dataset	CNN–LSTM multimodal temporal fusion	Accuracy 94.7%; precision 92.3%	Multimodal temporal models detect preclinical stress to enable early preventive actions
[194]	Thermal imaging pattern recognition	Free-range organic farms (small experimental group)	Template matching on thermograms	Sensitivity 95.1%, specificity 98.7%, detection ≈2 s	Fast detection for presence enables safe environmental treatments; demonstrates thermography in semi-outdoor contexts
[190]	Audio (MFCC) + CNN	Layers under controlled stressors	Acoustic classification of stress vocalizations	Classification accuracy 94%	Vocal biomarkers are non-contact indicators; they can complement thermal/enviro systems
[194]	YOLOv8 CV (feeder/waterer detection)	Small barns (≤15 hens)	Object detection + movement-based behavior inference	mAP@0.5 ≈91.5%; detection >92%	Low-cost CV is effective for feeding/drinking behavior monitoring—an early indicator of heat-related reduced intake
[203]	YOLOv8 + EMA attention + edge + digital twin	Caged layer farms; inspection robot	Improved small-target detection (beak abnormalities)	Detection accuracy 92.7%; mAP + 2.4% over baseline	Edge + digital twin supports real-time farm mapping; fine-feature detection aids health monitoring that flags stress-related feeding/beak issues
[191]	Low-cost IoT AMCU (T, RH, NH_3_, CH_4_)	Small-scale poultry lab tests (40–42 °C)	IoT + GSM + cloud; calibrated sensors	r > 0.96 vs reference; system cost ≈USD 76	Affordable continuous environmental sensing enables early warnings and potential auto-control in resource-limited settings
[204]	Cloud + IoT for gas monitoring (CO_2_ diurnal trends)	Poultry houses	Sensor feeds + ML predictions; dashboard & alerts	CO_2_ peak ≈570 ppm; significant diurnal pattern	Gas monitoring provides daily patterns linked to thermal load, useful for timing ventilation to reduce heat accumulation
[205]	Backpacks with monitoring devices + thermography	20 laying hens (welfare test)	Behavioral observations + thermography	Short-term preening ↑; eye temp ↑ on fit day; no long-term weight loss	Demonstrates the need for welfare checks and acclimation when using wearables on layers
[206]	Infrared thermography (head, body, shank)	864-layer chicks (1–42 days), four thermal regimes	Tukey test; BGHI assessment	Surface temp ↑ with ambient temp; optimal BGHI ≈ 66–82	Thermography is effective from early life; useful for pullet thermal management to prevent lifelong heat susceptibility.

T, temperature; RH, relative humidity; NH_3_, ammonia; CH_4_, methane; CO_2_, carbon dioxide; IoT, Internet of Things; AMCU, automated monitoring and control unit; GSM, Global System for Mobile Communications; ML, machine learning; RF, Random Forest; CNN, convolutional neural network; LSTM, long short-term memory; MFCC, Mel-frequency cepstral coefficients; YOLOv8, You Only Look Once version 8 object-detection algorithm; EMA, exponential moving average attention; CV, computer vision; mAP, mean average precision; BGHI, black globe humidity index; ↑ indicates increase.

### 4.4. Management and Welfare Practices

Effective management practices are central to maintaining welfare and productivity in layer chickens, particularly under heat stress [207]. Comprehensive welfare assessments show that welfare problems in laying hens, including bone lesions, integument damage, behavioral restriction, and injuries, are strongly influenced by the housing design, stocking density, and routine husbandry decisions [208]. Effective management practices are essential for reducing stress and maintaining welfare in layer chickens. Gentle, low-stress handling minimizes fear, injury, and corticosterone responses, while appropriate stocking densities reduce aggression, feather pecking, and productivity losses. Routine health inspections enable the early detection of disease or injury, limiting the duration of stress-related conditions, and staff trained in poultry behavior and welfare principles help maintain calm and consistent flock management [199,208]. Handling and transport are major sources of acute stress, and their effects vary among genetic lines [58]. Measures such as reducing the capture time, avoiding rough handling, optimizing crate space, ensuring adequate ventilation, and shortening transport duration can limit heat buildup, injury, and mortality [209]. Calm handling during routine procedures such as vaccination, beak trimming, and weighing further reduces stress responses and supports flock health and productivity.

### 4.5. Environmental Control

Laying hens strains have limited heat-dissipation capacity due to high metabolic heat production and dense feathering [210,211,212]. Consequently, maintaining house temperature and humidity within the thermoneutral range is essential for sustaining productivity and welfare during heat episodes.

Cooling systems directly reduce the thermal load and stabilize the THI. Studies show that cooled perches circulating chilled water lower core body temperature and behavioral stress responses during acute and chronic heat exposure, while fan–pad and fan–fogger evaporative systems reduce the shed temperature and oxidative stress, resulting in modest improvements in egg production [213]. Advanced evaporative technologies such as direct, indirect, and Maisotsenko-cycle systems can further reduce house temperature by 5–12 °C, depending on the humidity conditions [155].

Despite their effectiveness, evaporative cooling and negative-pressure ventilation systems require continuous energy input to operate circulation pumps, ventilation fans, and control units. The economic viability of these systems, therefore, depends on electricity costs, the flock size, and climatic conditions. Energy-efficient building design—including insulation, double-glazed openings, and hybrid renewable energy systems—has been proposed to substantially reduce overall energy requirements for environmental control in poultry facilities [156]. Ventilation design also strongly influences thermal uniformity. Novel negative-pressure systems and upward airflow displacement ventilation reduce temperature gradients, improve air distribution, and increase egg production compared with traditional tunnel ventilation [214,215]. For smaller farms and resource-limited production systems, simpler interventions may provide cost-effective alternatives to large mechanical cooling installations. For example, supplying cool drinking water during hot periods has been shown to maintain feed intake, egg production, and eggshell quality while reducing physiological stress indicators such as corticosterone levels in laying hens exposed to summer heat conditions [211]. Lighting management contributes indirectly by moderating activity and circadian rhythm stability, thereby reducing metabolic heat production during peak-heat periods.

Overall, integrated environmental strategies such as combining cooling, ventilation, air-quality control, and lighting management provide the greatest benefits for welfare and productivity. Evidence from multiple experimental and commercial studies summarized in Table 3 shows that these interventions reduce physiological stress indicators, stabilize behavior, and maintain egg production under thermal challenge. Future research should prioritize multi-system environmental trials, long-term welfare monitoring, and cost-effective designs suitable for diverse production systems.

**Table 3:** Environmental control strategies for heat stress mitigation in layer chickens: Effects on welfare and productivity.

References	Environmental Strategy	Technology/Intervention	Effect on Layer’s Welfare	Effect on Layer’ Productivity	Other Findings
[155]	Cooling systems	DEC, IEC, MEC advanced evaporative cooling	Lower thermal exposure time (ET), reduced THI by 3–10 units; improved thermal comfort	Not directly measured; improved thermal conditions imply productivity benefits	MEC performed best due to dew-point cooling; a low-cost alternative to AC
[214]	Ventilation optimization	Upward Airflow Displacement Ventilation (UADV)	More homogeneous air distribution; decreased heat stress by 9.4%; decreased winter cold stress by 68%	Indirectly improved performance by ensuring a uniform microclimate	Reduced pathogen spread via upward airflow; compatible with sensible cooling options
[216]	Air quality + temperature interaction	Control of cold stress + ammonia	Poor air quality worsens physiological stress	Reduced productivity and egg quality under cold stress interactions	Highlights the need for integrated management: temperature + air quality
[217]	Noise mitigation	Noise reduction strategies and soundproofing	Lower corticosterone; reduced fearfulness	Improved feed intake and egg output	Chronic noise (>85 dB) worsens HS-related stress

DEC, direct evaporative cooling; IEC, indirect evaporative cooling; MEC, Maisotsenko evaporative cooling; ET, exposure time; THI, temperature–humidity index; UADV, upward airflow displacement ventilation; AC, air conditioning; HS, heat stress; dB, decibels.

### 4.6. Genetic Traits

Genetic improvement represents a foundational strategy for mitigating heat stress in layer chickens because it provides cumulative, heritable gains that enhance thermal resilience, physiological stability, and long-term productivity under high ambient temperatures (Table 4). Indigenous and regionally adapted layer chicken breeds, Fayoumi, Benha line, and other native ecotypes, consistently show superior thermotolerance compared with commercial hybrids. Multi-generational selection in Fayoumi chickens demonstrated progressive improvements in egg production and eggshell strength under heat stress, accompanied by the upregulation of HSP90 and stress-response genes [218]. Similar patterns emerged in the Benha line and Fayoumi hens evaluated under increased temperatures, where the strong induction of HSP70, HSP25, BAG3, and metabolic regulators such as PDK4 supported a stable rectal temperature, body weight, and organ function during thermal challenge [219]. These responses reflect genetically encoded mechanisms involving protein stabilization, autophagy activation, metabolic adjustment, and oxidative damage mechanisms that enable chickens to preserve cellular integrity under heat load.

Evidence from long-term selection programs further demonstrates that thermotolerance can be improved through sustained genetic selection under field conditions. For example, selection programs in Fayoumi chickens exposed to thermal stress across multiple generations have shown progressive improvements in egg production and eggshell strength, while the magnitude of the heat-induced performance decline decreased from one generation to the next. These results indicate that genetic gains in both productivity and heat tolerance can be achieved simultaneously when selection pressure is applied under high ambient temperatures [220]. Similarly, quantitative genetic studies in Thai native chickens have shown that egg production traits under heat stress possess moderate heritability (h^2^ ≈ 0.15 ± 0.03), indicating that genetic improvement through selection is feasible. In these populations, estimated breeding values (EBVs) derived from temperature–humidity index (THI)-based selection models demonstrated measurable genetic progress in egg production when birds were selected for both productivity and heat tolerance simultaneously [139]. Long-term breeding programs in Thai native synthetic chicken lines evaluated over seven generations further confirm that genetic progress can be achieved under sustained thermal challenge. Multi-trait selection strategies targeting body weight, growth traits, and heat tolerance have demonstrated consistent genetic gains across generations, although improvements in growth traits were sometimes accompanied by reduced thermal resilience, highlighting the need for balanced breeding objectives [221].

Genome-wide association studies further demonstrate that thermotolerance is polygenic, with quantitative trait loci linked to body temperature regulation, metabolic pathways, and survival under heat challenge [222,223], supporting the use of genomic selection and marker-assisted breeding to improve resilience while maintaining production efficiency. Recent population genomic studies provide additional insight into the molecular basis of heat tolerance in indigenous chicken populations. Comparative genomic analyses of multiple indigenous ecotypes have identified several genomic regions under selection associated with thermoregulation and immune adaptation, including candidate genes such as HSP70, HSPA9, HSPH1, and HSP90AB1, which are involved in cellular stress responses and environmental adaptation (Table 4). These findings highlight the potential application of genomic selection strategies to accelerate the identification and propagation of heat-tolerant genotypes in poultry breeding programs [224]. Strain-specific differences in production resilience further demonstrate the genetic basis of thermotolerance. Fayoumi hens consistently exhibit minimal declines in egg quality and egg-shell characteristics under chronic heat exposure, whereas high-producing commercial lines such as Lohmann Brown show greater drops in shell thickness and egg weight.

However, genetic improvements for thermotolerance may involve trade-offs with productivity traits. Quantitative genetic analyses in native and synthetic chicken populations have revealed antagonistic genetic correlations between heat tolerance and growth-related traits, indicating that birds selected for rapid growth or high production efficiency may exhibit reduced resilience to thermal stress. For example, strong negative genetic correlations between growth traits and heat tolerance have been observed in tropical chicken lines, suggesting that selection for productivity alone can inadvertently reduce thermal adaptability [221].

Similarly, genetic correlation analyses between egg production and heat stress responses have demonstrated that increasing the environmental heat load is associated with reduced laying performance, with reported correlations between THI and egg production of approximately −0.306 [139]. These results illustrate the potential antagonistic relationship between productivity traits and heat tolerance, reinforcing the importance of incorporating thermal resilience into multi-trait breeding indices.

Strain comparisons also reveal differences between productivity and thermotolerance, as high-producing commercial lines often show larger declines in egg quality and shell characteristics during chronic heat exposure than physiologically robust native strains [225,226]. Genetic loci associated with bone mineral density and stress-response pathways may further enhance resilience when feed intake and mineral metabolism are disrupted by heat stress [218]. Despite progress, most genetic evaluations occur under controlled conditions, and few studies integrate thermotolerance with productivity and welfare traits in multi-trait breeding indices. Future studies should prioritize large-scale field validation, the crossbreed confirmation of candidate markers, and genomic strategies that simultaneously target thermoregulation, metabolic resilience, behavioral stability, and structural robustness to develop layer strains capable of sustaining productivity under increasingly variable climates.

**Table 4:** Genetic mechanisms and selection approaches for enhancing thermotolerance in layer chickens under heat stress conditions.

References	Genetic Focus	Findings	Mechanism of Heat-Stress Mitigation	Implications
[222]	Selection within Fayoumi	Improved egg production, stronger eggshells; ↑ HSP90	Enhanced cellular thermotolerance	Demonstrates rapid multi-generational genetic gain under heat
	GWAS under heat stress	QTL for BT, BW, and digestibility; moderate heritability	Polygenic regulation of thermoregulation	Enables marker-assisted and genomic selection
[219]	HSP and metabolic gene expression	Fayoumi and Benha lines show strongest HSP70, HSP25 responses	Stable cellular homeostasis under 32–40 °C	Local strains genetically superior for HSP-linked tolerance
[226]	Strain-specific oviductal gene expression	Fayoumi least affected; LB is the most sensitive	Expression shifts in GDF9, OC-17, HSP70 vary by tissue	Highlights tissue-specific adaptation patterns
[227]	SNP mapping under acute heat	80–440 SNPs linked to survival/ΔT	Genes regulate apoptosis and oxidative stress	Candidate markers for genomic selection
[225]	Thai native synthetic lines	Heritability drops 27–32% as THI ↑; Soi Nin is the most resilient	Productivity × thermotolerance antagonism	Importance of THI-informed evaluation

HSP, heat shock protein; GWAS, genome-wide association study; QTL, quantitative trait locus; SNP, single nucleotide polymorphism; THI, temperature–humidity index; BT, body temperature; BW, body weight; GDF9, growth differentiation factor 9; OC-17, ovocleidin-17; ΔT, change in temperature. ↑ indicates increase; × indicates interaction or trade-off.

## 5. Integrated Synthesis and Future Research Direction

**Figure 2:**
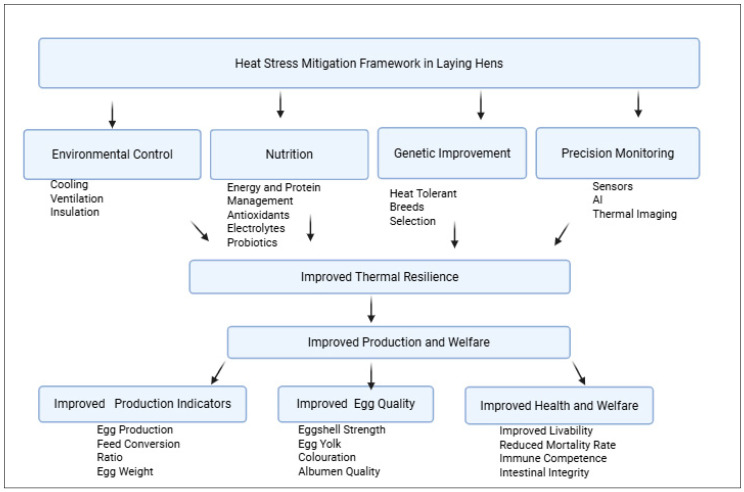
Heat stress mitigation framework in laying hens.

Heat stress in laying hens presents a complex multi-system biological disorder rather than a single physiological disturbance. Exposure to elevated ambient temperature simultaneously disrupts neuroendocrine regulation, metabolic homeostasis, oxidative balance, immune competence, and gastrointestinal integrity (Figure 2). These interconnected responses collectively determine the magnitude of productivity loss and welfare impairment observed in commercial layer systems (Figure 2). Activation of the hypothalamic–pituitary–adrenal (HPA) axis constitutes one of the earliest systemic responses to thermal stress. Elevated corticosterone levels redirect metabolic resources toward maintenance and survival functions, thereby suppressing feed intake, reproductive hormone secretion, and egg production. Concurrently, oxidative stress and mitochondrial dysfunction amplify cellular damage across metabolically active tissues such as the liver, kidney, and ovary. These changes are further compounded by the disruption of the intestinal barrier integrity and microbial homeostasis, which promotes endotoxin leakage and systemic inflammation. Importantly, these physiological pathways operate as a tightly coupled network in which dysfunction in one system propagates instability across others. The synthesis of available evidence further indicates that the effective mitigation of heat stress requires multi-level intervention strategies. Environmental modifications such as improved ventilation, evaporative cooling, and optimized housing design remain fundamental to reducing the thermal load. Nutritional strategies, including antioxidant supplementation, probiotics, and functional feed additives, can support intestinal health, immune competence, and metabolic stability under heat stress. In parallel, advances in genetic selection and breeding programs targeting thermotolerance provide long-term resilience by improving the physiological capacity of birds to withstand elevated temperatures. Emerging precision livestock farming technologies offer additional opportunities for proactive heat-stress management. Artificial intelligence, machine learning, and IoT-based sensor networks allow the continuous monitoring of environmental conditions, bird behavior, and physiological indicators. However, most current systems remain validated primarily under experimental conditions, and their performance in large-scale commercial poultry houses remains uncertain due to environmental variability, dust accumulation, sensor occlusion, and fluctuating ventilation patterns. Consequently, future research should prioritize large-scale validation trials and the integration of multimodal sensing systems capable of linking environmental monitoring with automated climate-control interventions.

Several key research gaps remain. First, most physiological studies focus on isolated stress pathways, whereas the integrated interactions among endocrine, metabolic, immune, and microbial systems remain insufficiently understood. Second, many mitigation studies are conducted under short-term experimental heat exposure, which may not accurately represent chronic thermal stress experienced in commercial production environments. Third, genetic improvement programs for thermotolerance require further evaluation under field conditions to quantify potential trade-offs between heat resilience and economically important production traits such as laying persistence, eggshell quality, and feed efficiency. Addressing these challenges will require interdisciplinary approaches that combine physiology, nutrition, genetics, engineering, and data science. Integrative research frameworks that link mechanistic biology with applied management strategies are essential to develop sustainable poultry production systems capable of maintaining productivity, animal welfare, and economic viability under increasingly variable climatic conditions.

The complex interaction of genetic, physiological, and environmental limitations is responsible for the impending difficulties in reducing heat stress for commercial layer chickens. A genetic–metabolic paradox, where high output directly clashes with heat tolerance, results from the newly developed laying hens’ high metabolic rates, which make them more susceptible to thermal distress as they are bred for peak egg production. Managing respiratory alkalosis and calcium depletion, which impair eggshell quality, while also avoiding the humidity trap, where conventional evaporative cooling fails in saturated air, presents a technical challenge for commercial farmers. Furthermore, at a time when international regulations are limiting the use of antibiotics, the immunosuppressive nature of heat stress increases disease susceptibility. Ultimately, the industry must balance the economic necessity of high-density housing with the rising demand for animal welfare, necessitating breakthroughs in precision nutrition, smart poultry housing climate technology, and the integration of thermotolerant genes into commercial lines to ensure long-term flock viability.

## 6. Conclusions

Heat stress remains a major constraint to layer chicken welfare and productivity, interacting with nutritional, environmental, behavioral, and genetic factors to disrupt physiology, reduce egg production and shell quality, and increase mortality risk. This review shows that heat stress is a multi-system challenge affecting neuroendocrine regulation, metabolism, oxidative balance, immunity, and gut integrity and therefore requires integrated mitigation strategies rather than isolated interventions. Evidence across physiology, precision monitoring, nutrition, and genetics demonstrates that early detection, improved housing environments, targeted dietary support, and selection for heat-tolerant genotypes can substantially improve resilience in modern layer systems. Advances in precision livestock technologies, genomic selection, and welfare-oriented management offer promising tools to address rising climate variability, but their effectiveness depends on large-scale validation, economic feasibility, and integration into practical farm management. Future research should prioritize multi-trait breeding for thermotolerance, productivity, scalable environmental solutions, and real-time monitoring systems linked to automated responses. Together, these approaches can support sustainable egg production while safeguarding hen welfare under increasingly challenging climatic conditions.

## Data Availability

All the data for this study are reported in this manuscript.
